# Revisiting the NMR Structure of the Ultrafast Downhill Folding Protein gpW from Bacteriophage λ

**DOI:** 10.1371/journal.pone.0026409

**Published:** 2011-11-04

**Authors:** Lorenzo Sborgi, Abhinav Verma, Victor Muñoz, Eva de Alba

**Affiliations:** 1 Centro de Investigaciones Biológicas, Consejo Superior de Investigaciones Científicas, Madrid, Spain; 2 Department of Chemistry and Biochemistry, University of Maryland, College Park, Maryland, United States of America; University of South Florida College of Medicine, United States of America

## Abstract

GpW is a 68-residue protein from bacteriophage λ that participates in virus head morphogenesis. Previous NMR studies revealed a novel α+β fold for this protein. Recent experiments have shown that gpW folds in microseconds by crossing a marginal free energy barrier (i.e., downhill folding). These features make gpW a highly desirable target for further experimental and computational folding studies. As a step in that direction, we have re-determined the high-resolution structure of gpW by multidimensional NMR on a construct that eliminates the purification tags and unstructured C-terminal tail present in the prior study. In contrast to the previous work, we have obtained a full manual assignment and calculated the structure using only unambiguous distance restraints. This new structure confirms the α+β topology, but reveals important differences in tertiary packing. Namely, the two α-helices are rotated along their main axis to form a leucine zipper. The β-hairpin is orthogonal to the helical interface rather than parallel, displaying most tertiary contacts through strand 1. There also are differences in secondary structure: longer and less curved helices and a hairpin that now shows the typical right-hand twist. Molecular dynamics simulations starting from both gpW structures, and calculations with CS-Rosetta, all converge to our gpW structure. This confirms that the original structure has strange tertiary packing and strained secondary structure. A comparison of NMR datasets suggests that the problems were mainly caused by incomplete chemical shift assignments, mistakes in NOE assignment and the inclusion of ambiguous distance restraints during the automated procedure used in the original study. The new gpW corrects these problems, providing the appropriate structural reference for future work. Furthermore, our results are a cautionary tale against the inclusion of ambiguous experimental information in the determination of protein structures.

## Introduction

The protein gpW from the *Escherichia coli* bacteriophage λ is a component of the viral particle that localizes in the connector between the head and tail [1]. Biochemical studies suggest that the role of gpW is to impede the exit of the pre-packaged DNA and organize the formation of the tail during virus assembly [2]. To perform these functions gpW is thought to participate in protein-protein and protein-DNA interactions. Such functional versatility makes gpW an interesting case example for studying macromolecular interactions, especially considering its small size (68 residues). The gpW 3D structure was originally determined by NMR using automatic assignment procedures [3]. This structure exhibits some peculiar features. For instance, the NMR study showed that gpW folds into an α+β topology consisting of two α-helices placed on top of a β-hairpin. Out of the 68 residues encoded by the gene only 50 form part of the folded structure, whereas three residues at the N-terminus and 15 at the C-terminus are disordered. The unstructured C-terminal fragment does not participate in stabilizing the native structure, but it is critical for connector assembly [1]. Another interesting structural property emerging from the NMR study is the conformation of the β-hairpin, which exhibits no significant twist and has both strands involved in tertiary contacts with the two helices, defining a single well-packed hydrophobic core according to the authors [3]. These features led to the classification of the gpW structure as a novel fold [3], status still valid today as manual and computational database searches fail to find any structural homologues.

The structural characteristics of gpW also make it an attractive candidate for folding studies. In fact, experimental studies of gpW's folding properties at the thermodynamic and kinetic level have been reported [1], [4]. Equilibrium denaturation experiments of gpW have shown that the same construct used for structural studies –i.e. includes a conservative Val→Thr mutation in position 2 and a C-terminal FLAG epitope followed by a hexa-histidine tag – is biologically active and folds-unfolds reversibly in a simple process compatible, at first glance, with the two-state folding mechanism [1]. A subsequent, more detailed, thermodynamic study of the gpW unfolding process using multiple structural probes and calorimetry demonstrated, however, that the α-helices of gpW melt at slightly higher temperature than the tertiary contacts defining the native environment of the sole tyrosine [4]. That is, the thermal unfolding of gpW is not concerted. In agreement with this observation, quantitative analysis of the DSC thermogram with the variable barrier model indicated that gpW folds over a marginal free energy barrier of ∼1 *RT*, which places this protein within the downhill folding regime [4]. Finally, kinetic analysis with the laser-induced temperature jump technique revealed that gpW is also an ultrafast folder, with a relaxation time of a few microseconds at its denaturation temperature [4]. These experimental studies have elicited the interest of theoretical-computational groups, which have simulated gpW folding using coarse-grained models [5], [6]. GpW has also become target for the extra-long molecular dynamics simulations performed by the Shaw group (K. Lindorff-Larsen, personal communication).

From the experimental side, the ultrafast folding kinetics and non-concerted unfolding behavior of gpW are the two exact properties required for performing an atom-by-atom analysis of protein folding [7]. In this analysis the thermal unfolding behavior of hundreds of individual atoms in the protein are monitored by NMR leading to a map of the folding interaction network of the protein [8]. However, before performing such analysis it is important to revisit the structural characterization of native gpW by NMR. This is so for several reasons. First, there are some differences between the original construct and that which was used for the multiprobe thermodynamic and kinetic studies. Particularly, the latter studies used a construct in which the original clone (including the Val→Thr mutation) was modified to remove the FLAG epitope, the histidine tag, and the last 6 C-terminal residues of the gpW gene, which were unstructured and faraway from the folded domain in the original structure [3]. The modifications are inconsequential in terms of thermal stability, as revealed by simple comparison between the unfolding curves monitored by far-UV CD on the two constructs [1], [4]. Nevertheless, it is useful to determine the NMR structure of the shorter construct for proper referencing of the atom-by-atom analysis. Second, the determination of the structure by multidimensional NMR using standard manual assignment would offer an opportunity to inspect the performance of the automatic methods that are being used in structural genomics projects [9]. Third, it is important to revisit the 3D structure of gpW given its novel fold and peculiar packing features.

Here we report the determination of the high-resolution structure of gpW without the C-terminal tags and unstructured residues using multidimensional NMR. We see that this structure conserves the overall α+β fold observed in the original study. However, the new structure shows clear differences in the packing of the β-hairpin against the two helices. In our structure the β-hairpin strands display the characteristic twist observed in other protein structures. The α-helices are less curved and rotated ∼40 degrees from one another relative to the original structure, thus forming a typical leucine zipper configuration. Further differences are found in tertiary packing, with the hairpin packing against the helices in an orthogonal rather than parallel orientation. These differences originate from the pattern of tertiary contacts observed among the aliphatic residues that conform the hydrophobic core. Comparison between the NMR datasets suggests that the structural discrepancies are caused by wrong long-range NOE assignments in the original study together with the inclusion of large sets of ambiguous NOEs in the automated structure calculation protocol. This interpretation is confirmed by molecular dynamics simulations in explicit solvent starting from both structures, and structure prediction calculations from the two sets of backbone chemical shift assignments using CS-Rosetta [10]. In fact, all these calculations converge onto a consensus structural ensemble for gpW that maintains the general structural features of our newly determined 3D structure. Therefore, this new structure should be used from now on as reference for future experimental and computational folding studies as well as for the interpretation of gpW's biological function.

## Results and Discussion

### New three-dimensional structure of gpW by NMR

**Figure 1:**
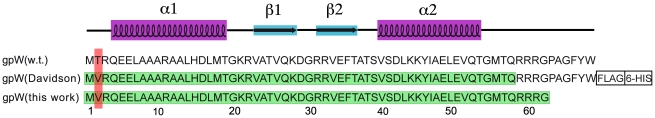
Amino acid sequence of wild type gpW and the different constructs used in the NMR studies. The secondary structure elements (helices in magenta and strands in cyan) are shown on top of the sequences. The conservative mutation is indicated in orange. Green bars mark the residues for which atomic coordinates have been reported from the previous NMR studies (PDB ID 1HYW) and this work (PDB ID 2L6Q).

**Figure 2:**
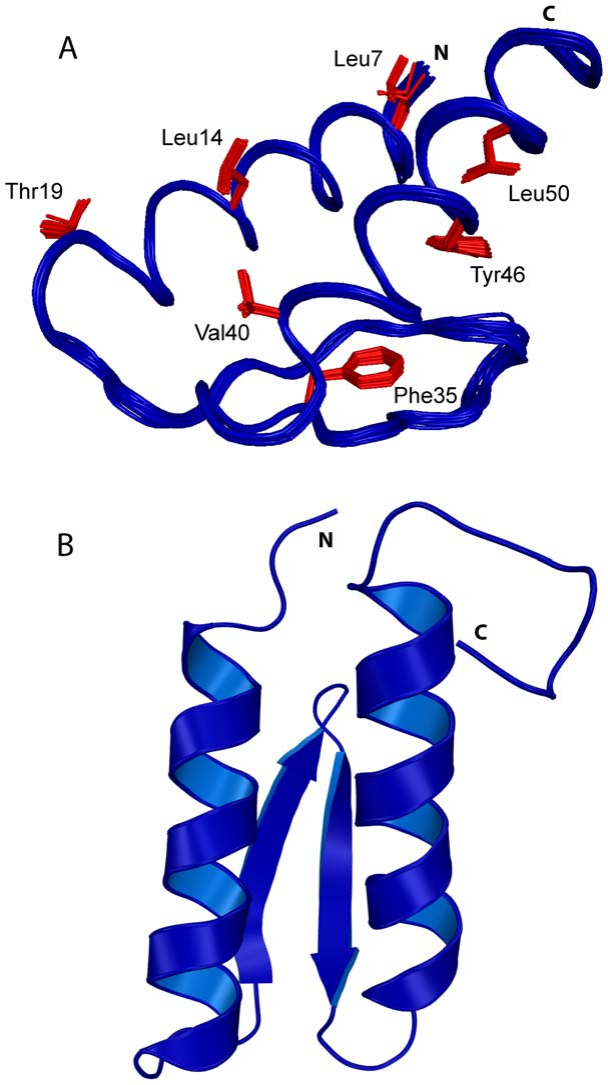
New NMR structure of gpW. (A) Backbone superposition of the 20 lowest energy conformers. Several side chains involved in hydrophobic contacts are shown in red. (B) Ribbon representation of the lowest energy structure.

We performed all NMR experiments on the same gpW construct that was used before for the multiprobe thermodynamic and kinetic analysis [4]. This construct was derived from the clone used in the original NMR study [1]. Thus, both proteins bear the same T2→V mutation that was included in the original study (note that this is the case even though the 1HYW pdb file, which corresponds to the prior structure, shows a threonine in position 2). The difference lies on the C-terminus, which has been shortened here to remove the FLAG epitope, the histidine tag, and six unstructured residues (**Fig. 1**). The new three-dimensional structure of gpW was determined with 723 unambiguous NOE-derived distances, together with 94 dihedral and 22 hydrogen bond restraints (coordinates deposited with the PDB accession code 2L6Q). The ensemble of 20 lowest energy conformers (**Fig. 2A**) does not show distance or angle restraint violations greater than 0.3 Å and 5°, respectively (**Table 1**). The ensemble of structures does not show significant deviations from covalent geometry and is well defined by the NMR data, as illustrated by the low root mean squared deviation (RMSD) calculated for the backbone and all heavy atoms (**Fig. 2A**) (**Table 1**). The C-terminal segment (residues 55–62) does not show NOE cross-peaks connecting them to the rest of the structure or NOE characteristic of secondary structure elements. In addition, residues 55–62 show chemical shifts close to random coil values. Altogether, the data indicate that this region is disordered, in agreement with the original structural study. The quality of the new gpW structure is high, with 91.2% of the residues (all those within the 4–54 structured segment) residing in the most favored regions of the Ramachandran plot for the whole ensemble (**Table 1**). The resolution of this NMR structure for gpW according to the program PROCHECK-NMR [11] is equivalent to an X-ray structure with a resolution between 1.0 Å and 1.8 Å (data not shown).

**Table 1:** Structural statistics of gpW*.

Restraints	r.m.s.deviations	
	20 lowest-energy conformers	Lowest energy conformer
**Distances, Å (723)**		
Intra-residue (156)		
Sequential |i−j| = 1 (140)		
Short-range |i−j|≤5 (187)	0.033±0.001	0.032
Long-range |i−j|≥5 (240)		
**Hydrogen bonds, Å (22)**	0.022±0.002	0.023
**Dihedrals (φ ϕ, °) (94)**	0.91±0.08	0.81
**Deviations form ideal covalent geometry**		
Bonds, Å	0.0033±0.0001	0.0031
Angles, °	0.38±0.01	0.36
Impropers, °	0.3±0.04	0.28
**Structure quality**		
Lennard-Jones potential	−204±6	−193
energy (Kcal mol^−1^)†		
**Ramachandran** #	Residues 4–54	
Residues in most	91.2%	
favored regions		
Residues in	8.8%	
disallowed regions		
**Coordinate precision, Å**	Residues 4–54	
Backbone heavy atoms	0.3±0.05	
All heavy atoms	1.0±0.07	

*Statistics were calculated for the 20 conformers with the lowest overall energies and no NOE or dihedral angle restraint violations greater than 0.3 Å and 5.0°, respectively.

† The Lennard-Jones van der Waals energy was calculated with the CHARMM PARAM19/20 parameters and was not included in structure calculation.

# Calculated with PROCHECK-NMR [11].

**Figure 3:**
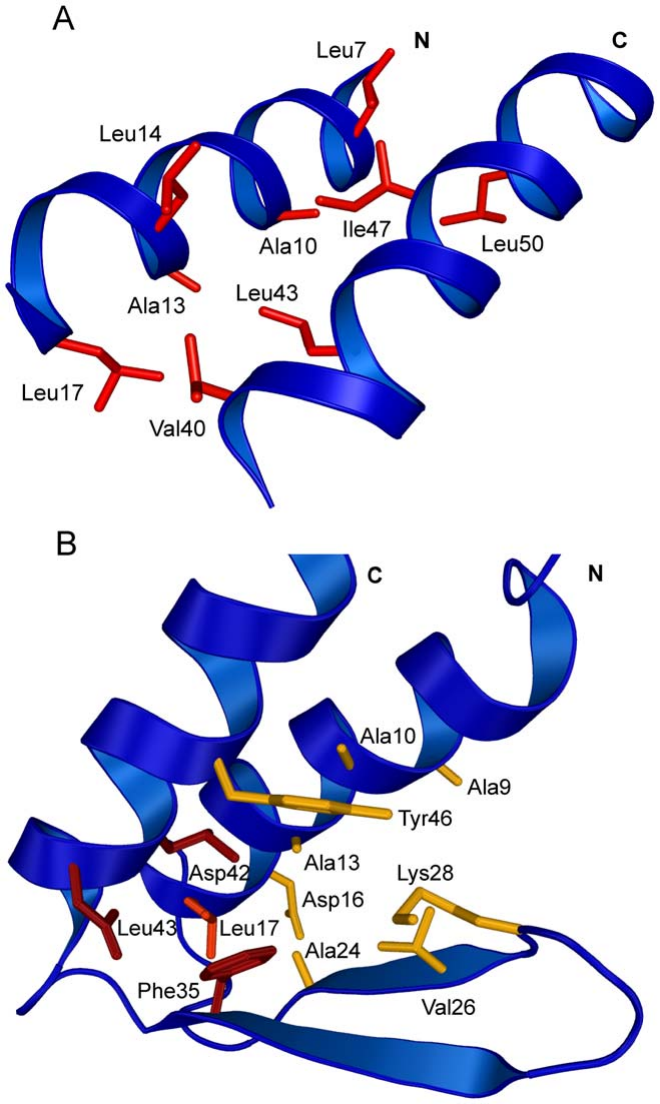
Detail on side chain packing in the new NMR structure of gpW. (A) Hydrophobic packing within helices involving numerous leucines. Residue name and number are indicated. (B) Hydrophobic packing of the helices and the β-hairpin. Residues responsible for helix contacts with strands 1 and 2 are illustrated in yellow and red, respectively. Leu 17, showing several contacts with strand 2, is highlighted in orange. Residue name and number are indicated.

**Figure 4:**
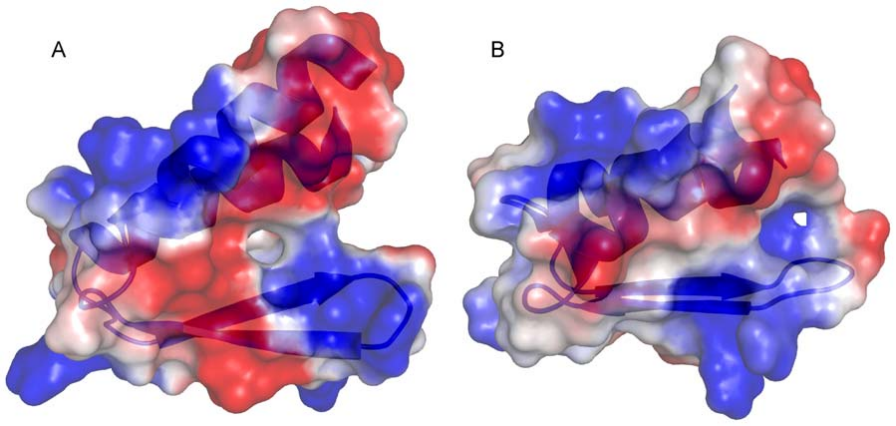
Electrostatic surface of gpW. Electrostatic surface of the new (A) and previous (B) NMR structures of gpW. Negatively and positively charged areas are shown in red and blue, respectively. The orientation of both structures is equivalent as shown by the ribbon diagram.

In terms of secondary structure, we see that the first helix of gpW starts in residue 4 and extends up to residue 19. A 3-residue turn connects helix 1 to the first β-strand (residues 23–28) followed by a β-turn and the second β-strand (residues 31–36), conforming a β-hairpin. Another 3-residue turn connects the second strand with α-helix 2 (residues 40–54) (**Fig. 2B**). In this gpW structure the β-hairpin displays the right-hand twist that is characteristic of antiparallel β-structures. Multiple long-range NOE cross-peaks connecting residues of the two helices were observed resulting in a very good definition of their relative position (**Fig. 2A**). These contacts (summarized in **Tables 2** **,** **3**) mostly involve amino acids on the hydrophobic face of the helices (**Fig. 3A** **,** **Table 2**). In contrast, fewer contacts were found between helical residues and the two strands. Helices 1 and 2 show several connectivities with strand 1 (**Table 3**), but only a few NOE cross-peaks were observed between strand 2 and either helix (most of them involve F35; **Fig. 3B** **,** **Table 3**). As a result, the new structure of gpW does not show a single well-packed hydrophobic core, but two orthogonal packing interfaces. One interface corresponds to the interactions that bring the two helices together to form a leucine zipper. The other interface corresponds to the interactions of the helices with only β-strand 1 of the β-hairpin, whereas strand 2 sits somewhat behind and is thus too far away to participate in tertiary contacts (**Fig. 3**). The electrostatic surface of the new gpW structure highlights the overall shape and charge distribution of the protein (**Fig. 4**). This calculation reveals that the slight opening between the helices and the β-hairpin is important to facilitate exposure to the solvent of several negatively charged side chains that would be buried on a more compact structure.

**Table 2:** Long-range (>*i, i+5*) NOEs connecting α-helix 1 and α-helix 2 in gpW*.

New structure	Previous structure
ARG 3	
LEU 50	X
GLN 53	X
THR 54	X
GLN 4	
X	LEU 50
X	GLN 53
THR 54	THR 54
X	MET 56
GLU 5	
X	THR 54
X	GLY 55
GLU 6	
LEU 50	X
LEU 7	
ILE 47	ILE 47
LEU 50	LEU 50
GLU 51	X
THR 54	X
ALA 8	
X	THR 54
ALA 10	
LEU 43	LEU 43
TYR 46	X
ILE 47	ILE 47
LEU 50	X
ARG 11	
X	LEU 43
X	LYS 44
ILE 47	ILE 47
X	GLU 51
ALA 13	
LEU 43	X
LEU 14	
VAL 40	VAL 40
LEU 43	LEU 43
LYS 44	LYS 44
ILE 47	ILE 47
HIS 15	
X	LYS 44
LEU 17	
VAL 40	VAL 40
X	LEU 43
MET 18	
VAL 40	X
LYS 44	X

*“X” denotes that the equivalent NOE was not observed.

**Table 3:** Long-range (>*i, i+5*) NOEs connecting α-helices and β-hairpin in gpW*.

New structure	Previous structure
LEU 7	
X	VAL 26
X	LYS 28
X	VAL 33
ALA 9	
VAL 26	X
ALA 10	
X	ALA 24
VAL 26	VAL 26
ALA 13	
ALA 24	X
VAL 26	X
LEU 14	
X	VAL 23
X	ALA 24
X	VAL 26
X	VAL 33
X	PHE 35
X	THR 36
ASP 16	
ALA 24	X
LEU 17	
VAL 23	VAL 23
ALA 24	ALA 24
PHE 35	PHE 35
THR 36	THR 36
ALA 24	
LEU 43	X
VAL 26	
LEU 43	LEU 43
TYR 46	X
LYS 28	
TYR 46	X
X	LEU 50
VAL 33	
X	LEU 43
PHE 35	
X	VAL 40
ASP 42	X
LEU 43	LEU 43

*“X” denotes that the equivalent NOE was not observed.

### Differences between the new and previous NMR structures

**Figure 5:**
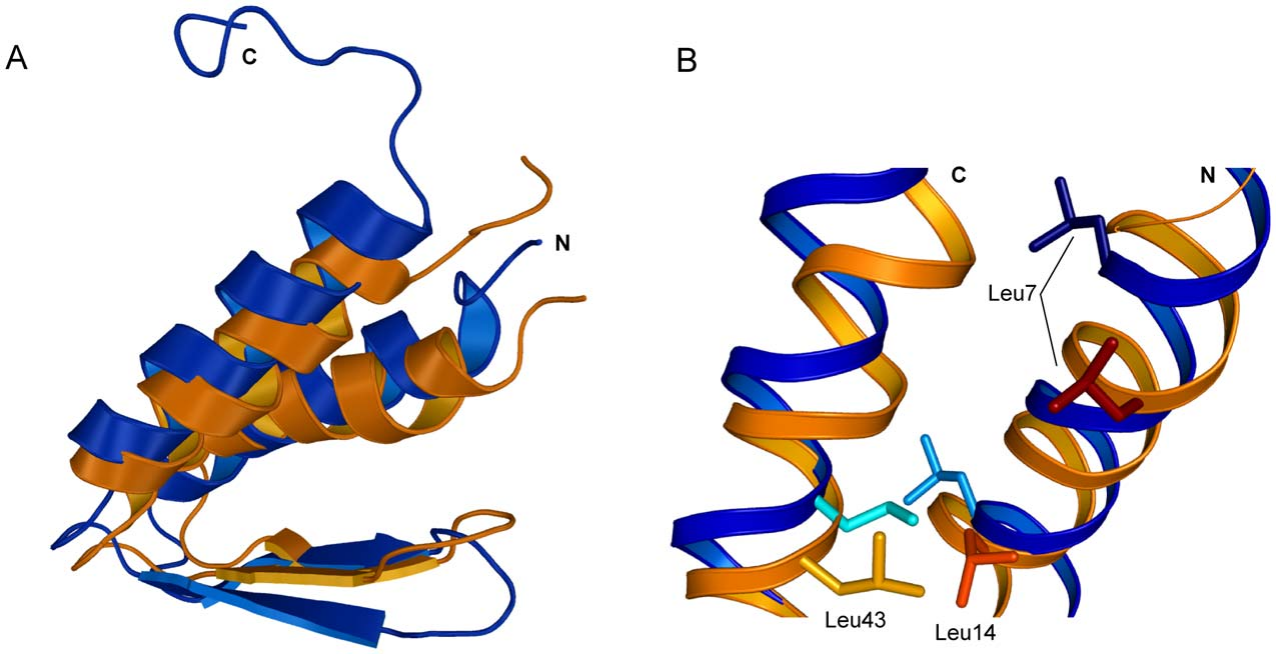
Differences between the new and previous NMR structures of gpW. (A) Ribbon diagram of the backbone superposition of the new (blue) and previous (orange) structures of gpW. (B) Detail on the different orientation of the side chain of Leu residues in the helices. Leu 7, Leu 14, Leu 43 in navy, sky blue, cyan for the new structure, and red, orange and light orange for the previous structure.

The two NMR structures of gpW are at considerable variance, and thus result in large backbone RMSD when superimposed (∼3 Å, **Fig. 5**). Specifically, the helices show different curvature and are rotated from one another as consequence of different interaction interfaces (**Fig. 5A,B**). In the new structure the hydrophobic side chains of the two helices are interlaced to form a leucine zipper (**Fig. 3A**) whereas in the original structure the hydrophobic side chains point towards the hairpin. The difference in β-hairpin orientation is also significant (**Fig. 5A**). All these differences originate from the fact that the pattern of tertiary contacts is drastically different in the two structures. The dissimilarity is particularly striking for the leucines in the helices: Leu 7 and Leu 14 in helix 1, and Leu 43 in helix 2 (**Fig. 5B**). Other residues in the helices, such as Ala 8, Ala 9, Ala 12 and Ala 13 in helix 1 and Ala 48 in helix 2 also adopt substantially different orientation. Altogether, these differences result in a different overall distribution of charges in the protein surface as illustrated in **Fig. 4**.

To investigate the source of these structural differences we need to consider several factors. A first consideration is the chemical changes between the constructs used in the two NMR studies (**Fig. 1**). In the previous work, a FLAG epitope followed by a hexa-histidine tag was added to the C-terminal of the gpW protein bearing the T2→V mutation. In this work the mutation is also present, but the tags are not and the sequence does not include the last 6 amino-acids encoded by the gene. However, according to the previous NMR studies, the unstructured natural tail, the FLAG epitope and the histidine tag do not show NOE contacts with the structured region. Accordingly, the coordinates deposited in the Protein Data Bank (PDB ID 1HYW) only span residues 1–58. Thus, the structural influence of the FLAG, histidine tag and the last C-terminal 10 residues should not be significant.

A second factor to consider is that the structures were determined under slightly different experimental conditions. There is some uncertainty in the experimental conditions used in the previous NMR study. The 1HYW PDB file sets the temperature employed for acquiring the NMR experiments at 25°C, whereas in the original article the experimental temperature is said to be 30°C. In the current work we determined the structure using NMR experiments acquired at 21°C. As a test, we acquired ^15^N-HSQC spectra [12] of gpW at 25°C and 30°C, and compared them with the chemical shift assignments of the previous study. We requested these assignments directly from Karen L. Maxwell and Alan R. Davidson because the Biological Magnetic Resonance Bank entry 3227 cited in the original NMR work [3] is empty. The comparison demonstrates that the changes in amide ^1^H^N^ chemical shifts are minimal, ruling out temperature as the source of the structural differences. The pH was identical (pH 6.5), but there is a difference in ionic strength: the previous NMR work used 200 mM sodium chloride and 10 mM phosphate buffer, whereas in this work the sample was prepared in 20 mM phosphate buffer. To investigate whether the difference in ionic strength could cause structural changes in gpW, we recorded a 4D-[^1^H-^13^C]-HMQC-NOESY-HSQC [13] experiment in a sample including 200 mM sodium chloride. This experiment reveals the exact same NOE pattern in the absence and in the presence of 200 mM salt (**Fig. S1**). Therefore, altogether these considerations indicate that the differences between gpW structures are not due to changes in experimental conditions.

The significant changes observed in the spatial arrangement of the secondary structure elements can only originate from substantially different sets of NOE-derived distance restraints. Comparing the NOE information from the current and previous (data also provided by Maxwell and Davidson) works we can note numerous differences, particularly relative to long-range contacts (**Tables 2** **,** **3**). Correct NOE cross-peak assignment crucially depends on the extent to which ^1^H, ^13^C and ^15^N chemical shifts are unambiguously assigned. In this regard, here we achieved 99% versus only 89%, in the previous work (not including the assignment of ^13^C carbonyl groups and side chain ^15^NH_2_ groups of the four Gln). In addition, we checked all chemical shift assignments with 4D-[^1^H-^13^C]-HMQC-NOESY-HSQC experiments [13], obtaining 98% of methyl ^13^C and ^1^H assignments in comparison to only 75% in the previous work. The correct assignment of methyl groups is essential to properly define the structure of the protein core.

For instance, in the previous NMR studies there are many NOEs assigned as involving Leu 14 with residues in the β-hairpin (**Table 3**) that are equally consistent with sequential or local NOEs. Specifically, the previous dataset includes NOEs connecting the C_δ_H_3_ groups of Leu 14 with the H^N^ of Ala 24 and the H^N^ of Phe35 (in front of Ala 24 in the hairpin). However, the chemical shifts of the C_γ_H_3_ groups of Val 23 are nearly identical to those of the C_δ_H_3_ moieties of Leu 14. It is not possible to distinguish between these different NOE assignments (i.e. Leu 14-Ala 24 or Val 23-Ala 24 (sequential), Leu 14-Phe 35 or Val 23-Phe 35 (local)) in the 3D ^15^N-edited-NOESY-type [12], [13] experiment performed by the authors of the previous studies. In such situation it is important to solve the ambiguity using, for example, 4D NOESY experiments, or alternatively to be conservative and avoid assigning ambiguous long-range NOE data. A similar scenario is found for several side chain to side chain long-range NOEs between the methyl groups of Leu 14 and the hairpin residues Ala 24, Val 33, and Phe 35 (**Table 2**). Here, in addition to Val 23, the chemical shift of one C_γ_H_3_ group of Val 26 (in the middle of the hairpin and in contact with Ala 24, Val 33 and Phe 35) is also nearly identical to one of the delta methyl moieties of Leu 14. Those contacts are most likely local. In fact, the putative long-range NOEs are not observed in the spectrum resulting from the 4D-[^1^H-^13^C]-HMQC-NOESY-HSQC experiment that we performed, where there is no ambiguity since the ^13^C chemical shifts of C_γ_H_3_ and C_δ_H_3_ groups of Val and Leu residues are different. In particular, the bottom left panel of **Fig. S1** illustrates the C_β_H_3_ plane of Ala 24 from a 4D-[^1^H-^13^C]-HMQC-NOESY-HSQC, which clearly shows that the NOEs with the side chain of Leu 14 (included in the distance restraint list of the previous work) are not observed. The bottom right panel (**Fig. S1**) shows similar discrepancies for the C_α_H_α_ plane of Leu 7, for which there are no observable NOE connectivities with the side chain of Val 26 in contrast with the previous distance restraint dataset and structure.

According to the NOE data provided by the authors of the previous study, both sets of potential NOE assignments (sequential and long-range) were included in the structure calculation listed as unambiguous. Moreover, the previous structure was calculated adding on top many other NOEs classified by the authors as ambiguous, making up to ∼25% of the total distance restraints in the final structure calculation round [3]. In this work we have used the standard manual procedure and have used only unambiguous NOE information from 3D and 4D experiments, resulting in a total of 723 distance restraints. As a result of the different NMR datasets and the two strategies for structure calculation the quality of the previous and new structures is drastically different. This is easily demonstrated by comparing the scores of standard structure quality checks. For example, MolProbity [14] indicates that the new gpW structure (PDB ID 2L6Q) is in the 27^th^ percentile, whereas the previous one (PDB ID 1HYW) is only in the 1^st^ percentile.

### Computational tests on the 1HYW and 2L6Q structures of gpW

From an NMR viewpoint, the new structure (PDB ID 2L6Q) is the one that best represents the native conformational ensemble of gpW, and the one that should be used from now on because it is based on a much more complete experimental dataset and does not use ambiguous NOE-derived restraints. However, the differences between the two structures raise an interesting surrogate question: which of these structures is more in accordance with what we know about protein three-dimensional structure, stability and folding? This is a particularly interesting question given the novel fold features embodied in the gpW structures. To address this question we performed molecular dynamics simulations in explicit solvent as well as structure-prediction calculations from backbone chemical shifts using the CS-Rosetta program [10].

**Figure 6:**
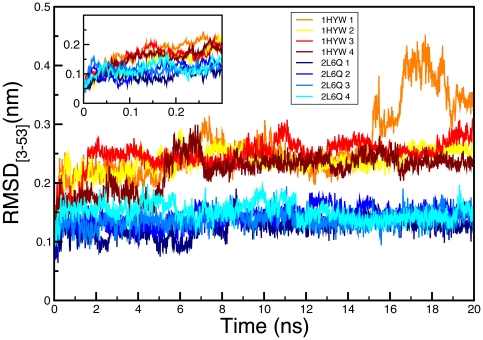
Molecular dynamics simulations of gpW starting from 2L6Q and 1HYW structures. RMSD as a function of time of the four MD simulations starting from our new gpW structure (2L6Q; shades of blue) and from the original structure (1HYW; shades of red). The inset is a blowup of the first 0.3 nanoseconds of the MD trajectories.

To have sufficient statistical sampling we performed four independent molecular dynamics simulations starting from the atomic coordinates of either 1HYW or 2L6Q. These simulations were carried out using the CHARMM27 force-field with the TIP3P water model and were run for 20 ns to produce a total of 80 ns simulation time for each of the two starting structures. The four 20 ns trajectories starting from 2L6Q resulted in a fast (<1 ns) relaxation to an ensemble of structures with ∼1.5 Å backbone RMSD (computed for residues 3–53) and stayed within that ensemble for the rest of the trajectory (**blue in** **Fig. 6**). The average backbone RMSD for the last 5 ns of the four trajectories was 1.42 Å, indicating that the MD ensemble is for all practical purposes equivalent to the initial 2L6Q structure. This result indicates that 2L6Q is a stable and robust structure for gpW according to the CHARMM27 force-field. We observed a quite different behavior for the simulations starting from 1HYW (**red in** **Fig. 6**). In this case, the fast <1 ns relaxation resulted in significantly larger deviations from the starting structure, leading to over 2.5 Å backbone RMSD (again computed for residues 3–53). In one of the four simulations we also observed a second structural transition at ∼16 ns to an ensemble ∼4 Å RMSD away from the starting structure. The remaining three simulations did not show this transition, but since this transition happened near the end of the simulation it is possible that the others would have undergone the same change had their simulation time been longer. In any event, the average backbone RMSD relative to 1HYW for the last 5 ns of the four trajectories is 2.75 Å, which is much higher than what we observed in the 2L6Q simulations. This result indicates that the 1HYW structure is more energetically strained.

**Figure 7:**
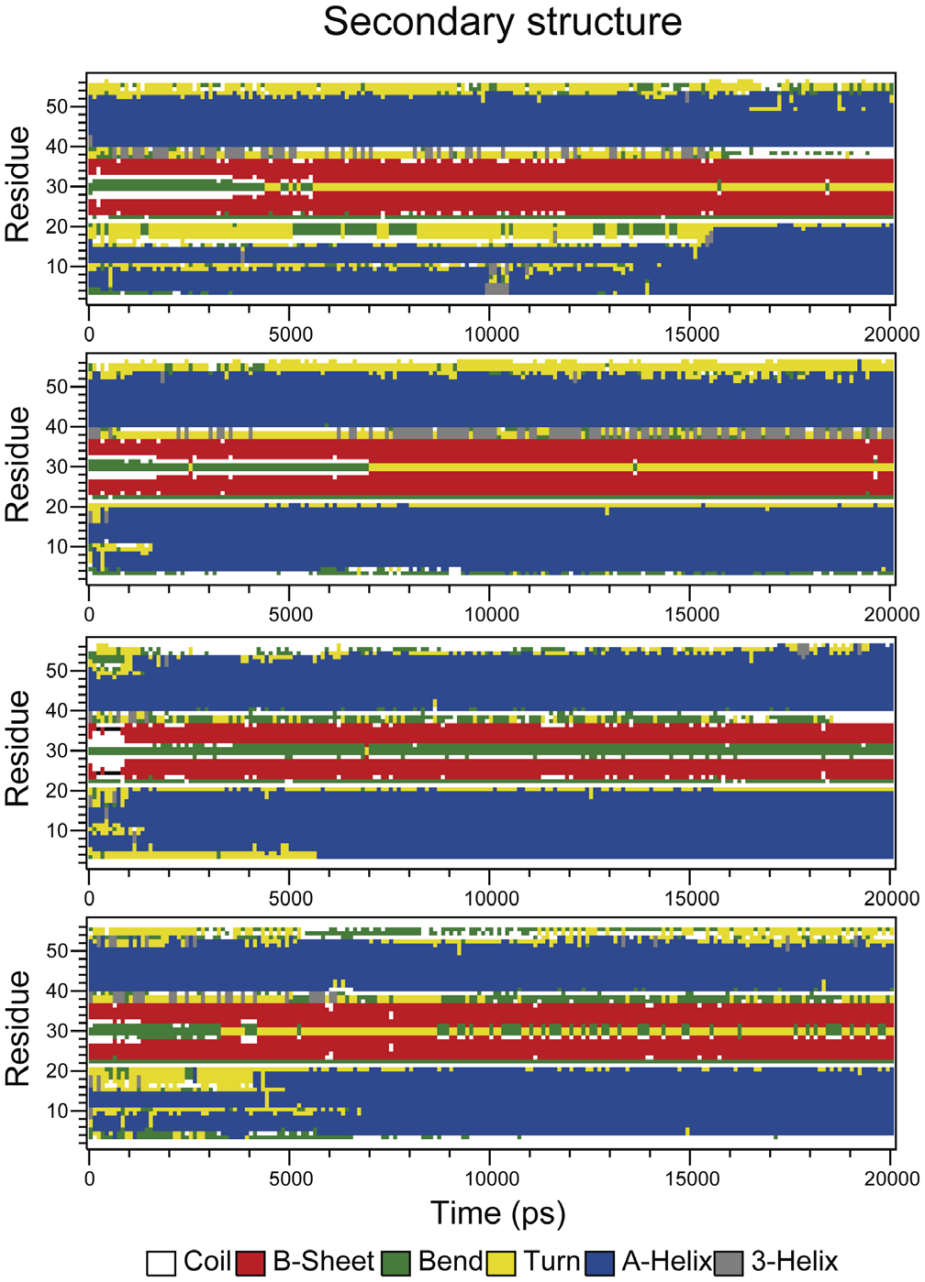
Changes in the secondary structure of gpW during the MD simulations starting from 1HYW. The panels show the secondary structure assignment of the 58 structured residues of 1HYW as a function of time for the four 20 ns long MD simulations. The color code is displayed in the figure.

However, the most interesting result emerging from the 1HYW MD trajectories is that, as they wander off the initial structure, the ensemble becomes more similar to the 2L6Q structure. The average backbone RMSD for the last conformation of the four trajectories is 2.31 Å relative to the 2L6Q structure, compared to 2.98 Å relative to the starting 1HYW structure. In other words, the MD simulations relax the strain contained in the 1HYW structure leading to a final ensemble that converges onto the new 2L6Q structure. The representation of the secondary structure as function of time for the 1HYW simulations shows that the structural changes occurring during the first 2 ns involve mostly the hairpin and the N-terminal helix (**Fig. 7**). The central region of the hairpin changes from a bend to a more typical β-turn (as seen in 2L6Q), and the strands adopt a twisted conformation in which the second strand moves away from the helices (**Fig. S2**). Moreover, the N-terminal α-helix becomes longer, as it is observed in the 2L6Q structure (**Fig. 7**).

**Figure 8:**
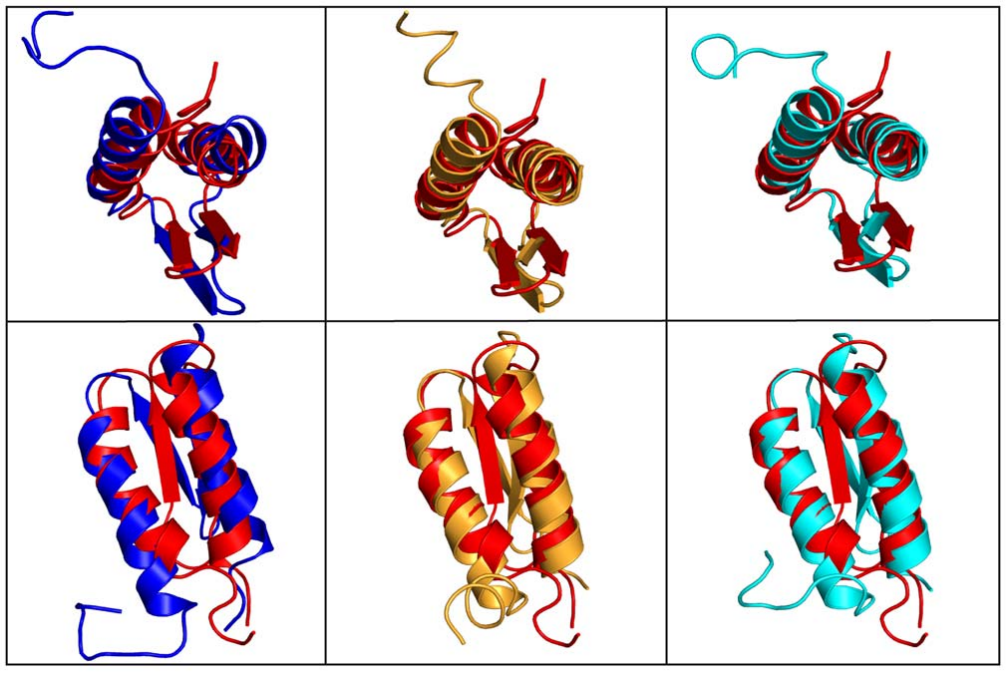
Comparison of experimentally determined 3D structures of gpW and CS-Rosetta predictions. The panels show pairwise superpositions of the original experimental gpW structure (1HYW; dark red) with the new experimental structure (2L6Q; dark blue) and the CS-Rosetta predictions using the original backbone chemical shift assignments (orange) and the chemical shift assignments obtained here (cyan). The upper and lower rows show two different orientations.

The same overall conclusion emerges from the structure prediction calculations using the backbone chemical shift assignments as input data for CS-Rosetta [10]. In this case, the predicted native structure for gpW is nearly identical, whether we introduce the original or our new backbone chemical shift table as input data. The fact that the two predictions are essentially the same structure confirms that the differences between the two experimentally determined structures do not originate from discrepant backbone assignments, but are caused by the list of long-range NOEs used as distance restraints, as discussed in the previous section. The two predicted structures are very similar to 2L6Q (1.27 and 1.47 Å backbone RMSD) and much less to 1HYW (2.56 and 2.55 Å backbone RMSD). Moreover, pairwise structural comparisons show that the CS-Rosetta predicted structures share the same distinctive structural characteristics of 2L6Q (**Fig. 8**). The β-hairpin has a clear right-hand twist and is placed orthogonally to the α-helical interface so that only strand one is in direct contact with it. In contrast, in 1HYW the β-hairpin is flat and packing in parallel to the helical interface (upper row in **Fig. 8**). By the same token, the two helices are straighter, follow a leucine zipper arrangement, and the N-terminal helix is longer, exactly as observed in 2L6Q (lower row in **Fig. 8**).

MD simulations and CS-Rosetta predictions provide complementary computational tests for the structures because the two calculations are based on completely different input data and methodology. MD simulations use the atomic coordinates from the PDB file whereas CS-Rosetta uses the chemical shift data directly. Thus, for one calculation the input data are affected by the distance restraint information and structure determination procedure, but for the other calculation are not. Another critical difference is that the MD simulations provide dynamical information by solving Newton's equation of motion. CS-Rosetta, on the other hand, is a prediction algorithm based only on energy minimization. Finally, the CHARMM27 and Rosetta force-fields are drastically different. Therefore, the fact that both methods produce structures-ensembles that converge to the main characteristics of the 2L6Q structure regardless of the dataset that is used as input in the calculation indicates that our gpW structure is in accord with what is known about protein structure, folding and stability. These computational tests confirm that the 2L6Q structure corrects the packing issues that are present in 1HYW and which led to a distorted hairpin conformation and helix-helix interaction interface, as well as the partial burial of negative charges (**Fig. 4**).

## Materials and Methods

### Gene Cloning and plasmid construction

Residues 1–62 of the original gene of gpW provided by Alan R. Davidson was subcloned into the expression vector pBAT [15]. The new construct bears the same T2→V mutation present in the original construct, but lacks the purification tags (FLAG epitope and Histidine tag) and the last six unstructured residues of gpW (**Fig. 1**).

### Protein Expression and Purification

The 62-residue protein gpW was expressed in BL21(DE3) E. Coli strain (Novagen). Bacteria were grown at 37°C. After reaching an OD_600_ of 0.6–0.7, protein expression was induced by adding 1 mM IPTG (isopropyl-β-D-thiogalacto-pyranoside). Expression continued for 4 h at 37°C. The cells were harvested by centrifugation and resuspended in a 20 mM phosphate buffer at pH 6.0, containing 0.2 mM NaCl and 0.1 mM protease inhibitor cocktail (Sigma). Cells were then lysed by sonication at 4°C and centrifuged at 25000 rpm for 30 minutes. The supernatant was purified by cation exchange chromatography using an SP sepharose Fast Flow column (GE Healthcare). A second purification step was necessary using reverse phase chromatography with a gradient of 0–95% water/acetonitrile and 0.1% trifluoroacetic acid. The purified protein solution was lyophilized and analyzed by SDS-polyacrylamide gel electrophoresis and electrospray mass spectrometry. Uniformly ^15^N- and ^13^C-labeled gpW was produced using ^13^C_6_-D-glucose and ^15^NH_4_Cl (Spectra Stable Isotopes) as sole carbon and nitrogen sources, respectively.

### NMR spectroscopy

NMR samples were prepared at 1 mM ^13^C, ^15^N-labeled gpW in 20 mM phosphate buffer, 0.1 mM NaN_3_, pH 6.5, 5% D_2_O/H_2_O and 100% D_2_O. Under these conditions the 62-residue gpW remains soluble and monomeric according to NMR linewidth values. NMR experiments were acquired at 294 K in a Bruker Avance III 600 MHz spectrometer equipped with a triple resonance triaxial-gradient probe. Sequence backbone chemical shift assignments were obtained from the experiments; [^1^H-^15^N]-HSQC, 3D HNCACB and 3D CBCA(CO)NH [12], [13]. Side chain ^1^H, ^15^N and ^13^C assignments were obtained from 3D HBHA(CO)NH, 3D CC(CO)NH and 3D HCCH-TOCSY [12], [13]. NOE data were obtained from 3D-^15^N-[^1^H-^1^H]-NOESY (110 ms mixing time) and 4D-[^1^H-^13^C]-HMQC-NOESY-HSQC (110 ms mixing time) [12], [13]. All experiments were processed with NMRPipe [16] and analyzed with PIPP [17].

### Structure calculation

Peak intensities from NOESY experiments were translated into a continuous distribution of interproton distances. Errors of 25% of the distances were applied to obtain lower and upper distance limits. A total of 723 unambiguous interproton distance restraints were used. 22 hydrogen bond distance restraints (r_NH-O_ = 1.9–2.8 Å, r_N-O_ = 2.8–3.4 Å) were defined according to the experimentally determined secondary structure of the protein. The program TALOS+ [18] was used to obtain 94 φ and ϕ backbone torsion angle constraints for those residues with statistically significant predictions. Structure calculations were performed with and without intra-residue distance restrains. However, the resulting structures did not show any substantial differences. Structures were calculated with the program X-PLOR-NIH 2.16.0 [19]. The starting structure was heated to 3000 K and cooled in 30,000 steps of 0.002 ps during simulated annealing. The minimized target function includes a harmonic potential for experimental distance restraints, a quadratic van der Waals repulsion term for the non-bonded contacts, a square potential for torsion angles and a torsion angle database potential of mean force. The final ensemble of 20 NMR structures was selected based on lowest energy and no restraint-violation criteria. These conformers have no distance restraint violations and no dihedral angle violations greater than 0.3 Å and 5°, respectively. Structures were validated using PROCHECK-NMR [11] and MolProbity [14], which show that the family of 20 structures is of considerably high quality in terms of geometry and side chain packing. Structures were analyzed with PYMOL [20]. Coordinates were deposited in the Protein Data Bank with accession code 2L6Q and chemical shifts were deposited in the Biological Magnetic Resonance Bank (BMRB code 17321).

### Molecular Dynamics Simulations

Molecular dynamics simulations were performed using GROMACS [21] version 4.5.3 using the CHARMM27 force field [22]. The protein structures were placed in a cubic unit cell with a minimum distance of 1.0 nm to the box edge. Steepest descent minimization was performed followed by an addition of ions in order to neutralize the system, yielding a final system of about 6,800 TIP3P water molecules and 1 Cl^−^ ion for 1HYW and 10,000 TIP3P water molecules and 4 Cl^−^ ions for 2L6Q. The simulation box of 2L6Q is larger due to the presence of a longer C-terminal tail. Another steepest descent minimization was performed to the solvated system. Non-bonded interactions were evaluated using a twin range cutoff of 0.9 and 1.4 nm together with a reaction field (RF) correction [23] to compensate for the neglect of electrostatic interactions beyond the longer range cutoff (ε_RF_ = 78.0). Interactions within the shorter-range cutoff were evaluated at every step (2 fs), whereas interactions within the longer-range cutoff were evaluated every 5 steps (10 fs). Temperatures were maintained using the Berendsen thermostat [24] with a coupling time of 0.1 ps. The Berendsen barostat was used with a coupling time of 1 ps and an isotropic compressibility of 4.5×10^−5^bar^−1^ to maintain a constant pressure of 1bar. All bonds were constrained using the LINCS algorithm [25]. Periodic boundary conditions were applied and simulations were run using a 2 fs time step, with neighbor list updates every 10 fs. Each system was first energy-minimized and then simulated for 2.5 ps with position restraints on all heavy atoms to relax the system. After a further 5 ps of simulation without restraints, 20 ns production runs were performed. Four 20 ns-long simulations were performed for both 1HYW and 2L6Q.

### Structure Predictions using CS-Rosetta

Calculations using CS-Rosetta [10] were performed using the experimentally determined ^13^C_α_, ^13^C_β_, ^15^N, ^1^H_α_ and ^1^H^N^ NMR chemical shifts. For the calculations we used either the chemical shifts from the original NMR study (provided directly by K. L. Maxwell and A. R. Davidson), or the ones determined by us in this study (available in BMRB 17321). 1200 structural models were generated with CS-Rosetta for each chemical shift dataset and the lowest energy structure was chosen as the final prediction.

## Supporting Information

Figure S1 **Slices of 4D-[^1^H-^13^C]-HMQC-NOESY-HSQC spectra of gpW.** Spectra were acquired at pH 6.5, 20 mM phosphate (blue spectrum) and 200 mM NaCl (red spectrum). The corresponding residue and ^13^C-^1^H pair are shown on top of each panel. The NOE cross-peak assignments are indicated. Empty squares in the bottom panels indicate the position of NOE cross-peaks corresponding to several distance restraints used in the previous structure calculation (**Tables 2** **,** **3**) that were not observed in our spectra.
(TIF)

Figure S2 **GpW structures from NMR and MD simulations.** Backbone superposition of the previous gpW NMR structure (red; PDB ID 1HYW), the new (blue; PDB ID 2L6Q) and the structures resulting from the four molecular dynamics simulations on PDB ID 1HYW shown in **Fig. 6** (all in orange).
(TIF)

## References

[1] Maxwell KL, Davidson AR, Murialdo H, Gold M (2000). Thermodynamic and functional characterization of protein W from bacteriophage lambda. The three C-terminal residues are critical for activity.. J Biol Chem.

[2] Murialdo H, Xing X, Tzamtzis D, Haddad A, Gold M (2003). The product of the bacteriophage lambda W gene: purification and properties.. Biochem Cell Biol.

[3] Maxwell KL, Yee AA, Booth V, Arrowsmith CH, Gold M (2001). The solution structure of bacteriophage lambda protein W, a small morphogenetic protein possessing a novel fold.. J Mol Biol.

[4] Fung A, Li P, Godoy-Ruiz R, Sanchez-Ruiz JM, Muñoz V (2008). Expanding the realm of ultrafast protein folding: gpW, a midsize natural single-domain with alpha+beta topology that folds downhill.. J Am Chem Soc.

[5] de Sancho D, Rey A (2008). Energy minimizations with a combination of two knowledge-based potentials for protein folding.. J Comp Chem.

[6] Bruscolini P, Naganathan AN (2011). Quantitative prediction of protein folding behaviors from a simple statistical model.. J Am Chem Soc.

[7] Sadqi M, Fushman D, Muñoz V (2006). Atom-by-atom analysis of global downhill protein folding.. Nature.

[8] Muñoz V, Sadqi M, Naganathan AN, de Sancho D (2008). Exploiting the downhill folding regime via experiment.. HFSP Journal.

[9] Moseley HN, Montelione GT (1999). Automated analysis of NMR assignments and structures for proteins.. Curr Opin Struct Biol.

[10] Shen Y, Vernon R, Baker D, Bax A (2009). De novo protein structure generation from incomplete chemical shift assignments.. J Biomol NMR.

[11] Laskowski RA, Rullmannn JA, MacArthur MW, Kaptein R, Thornton JM (1996). AQUA and PROCHECK-NMR: programs for checking the quality of protein structures solved by NMR.. J Biomol NMR.

[12] Bax A, Grzesiek S (1993). Methodological advances in protein NMR.. Accounts Chem Res.

[13] Cavanagh J, Fairbrother WJ, Palmer AG(3rd), Rance M, Skelton NJ (2006).

[14] Davis IW, Leaver-Fay A, Chen VB, Block JN, Kapral GJ (2007). MolProbity: all-atom contacts and structure validation for proteins and nucleic acids.. Nucleic Acids Res.

[15] Peranen J, Rikkonen M, Hyvonen M, Kaariainen L (1996). T7 vectors with modified T7lac promoter for expression of proteins in Escherichia coli.. Anal Biochem.

[16] Delaglio F, Grzesiek S, Vuister GW, Zhu G, Pfeifer J (1995). NMRPipe: a multidimensional spectral processing system based on UNIX pipes.. J Biomol NMR.

[17] Garrett DS, Powers R, Gronenborn AM, Clore GM (1991). A common sense approach to peak picking two-, three- and four-dimensional spectra using automatic computer analysis of contour diagrams.. J Magn Reson.

[18] Shen Y, Delaglio F, Cornilescu G, Bax A (2009). TALOS+: a hybrid method for predicting protein backbone torsion angles from NMR chemical shifts.. J Biomol NMR.

[19] Schwieters CD, Kuszewski JJ, Tjandra N, Clore GM (2003). The Xplor-NIH NMR molecular structure determination package.. J Magn Reson.

[20] DeLano (2004).

[21] Hess B, Kutzner C, van der Spoel D, Lindahl E (2008). GROMACS 4: Algorithms for highly efficient, load-balanced, and scalable molecular simulation.. J Chem Theor Comp.

[22] Bjelkmar Pr, Larsson P, Cuendet MA, Hess B, Lindahl E (2010). Implementation of the CHARMM force field in GROMACS: Analysis of protein stability effects from correction maps, virtual interaction sites, and water models.. J Chem Theor Comp.

[23] Tironi IG, Sperb R, Smith PE, van Gunsteren WF (1995). A generalized reaction field method for molecular dynamics simulations.. J Chem Phys.

[24] Berendsen HJC, Postma JPM, van Gunsteren WF, DiNola A, Haak JR (1984). Molecular dynamics with coupling to an external bath.. J Chem Phys.

[25] Hess B, Bekker H, Berendsen HJC, Fraaije JGEM (1997). LINCS: A linear constraint solver for molecular simulations.. J Comp Chem.

