# Supplementary figures and images for: Revisiting the NMR Structure of the Ultrafast Downhill Folding Protein gpW from Bacteriophage λ
Source: PLoS One. 2011 Nov 4;6(11):e26409. doi: 10.1371/journal.pone.0026409 (PMC3208555; doi:10.1371/journal.pone.0026409)

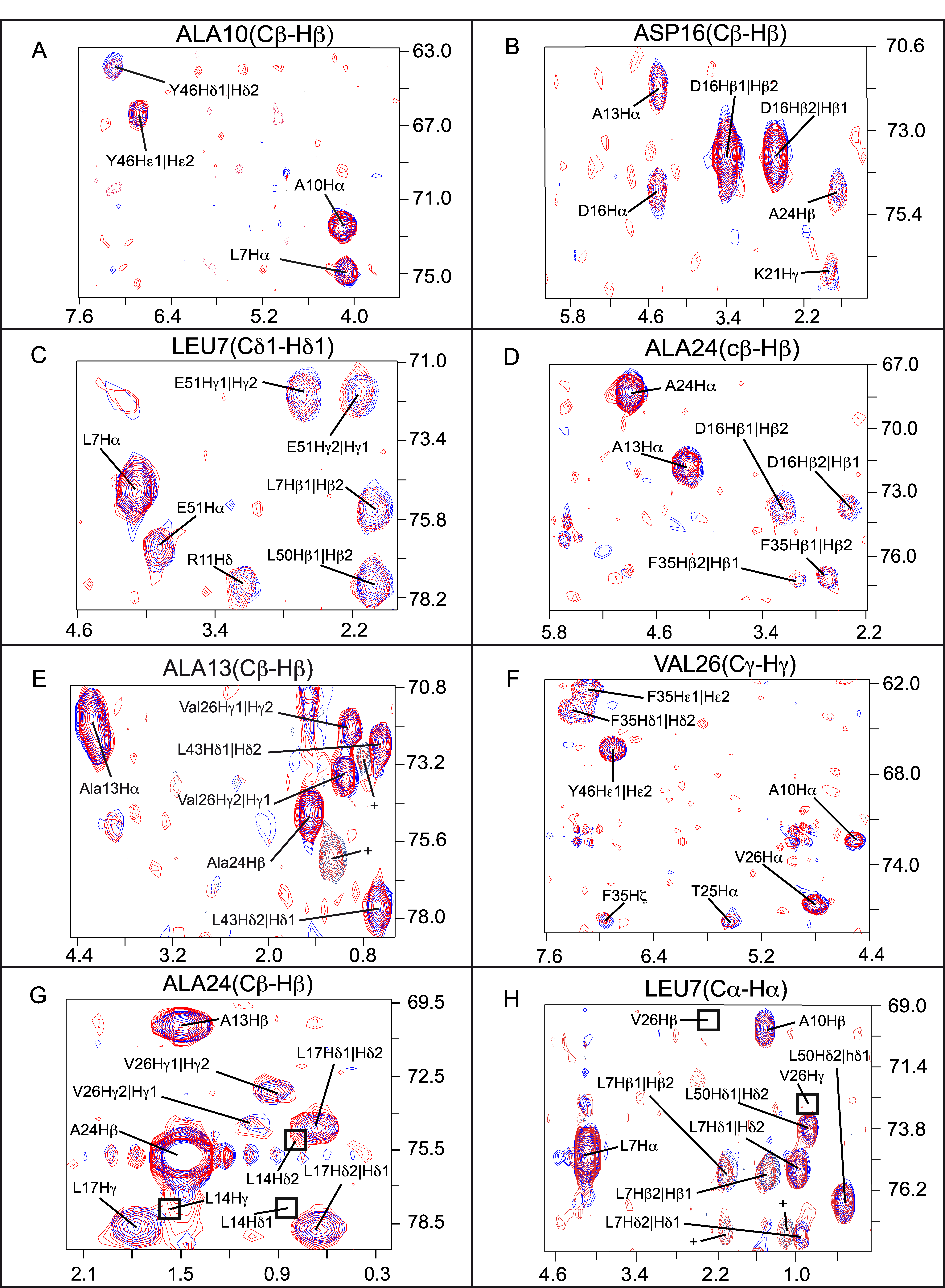

Supplement: Figure S1 — Slices of 4D-[1H-13C]-HMQC-NOESY-HSQC spectra of gpW. Spectra were acquired at pH 6.5, 20 mM phosphate (blue spectrum) and 200 mM NaCl (red spectrum). The corresponding residue and 13C-1H pair are shown on top of each panel. The NOE cross-peak assignments are indicated. Empty squares in the bottom panels indicate the position of NOE cross-peaks corresponding to several distance restraints used in the previous structure calculation ( Tables 2 , 3 ) that were not observed in our spectra. (TIF) [file pone.0026409.s001.tif]

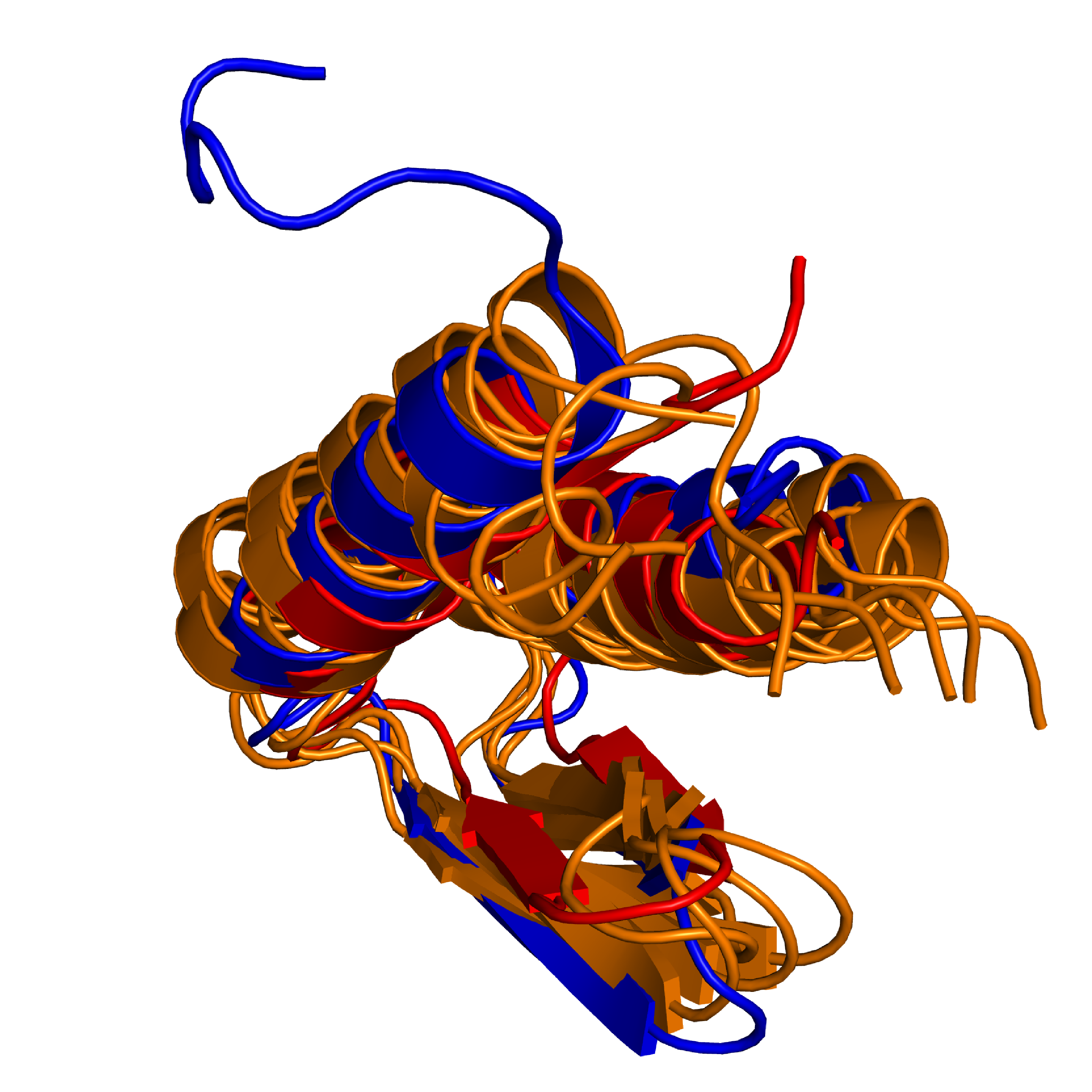

Supplement: Figure S2 — GpW structures from NMR and MD simulations. Backbone superposition of the previous gpW NMR structure (red; PDB ID 1HYW), the new (blue; PDB ID 2L6Q) and the structures resulting from the four molecular dynamics simulations on PDB ID 1HYW shown in Fig. 6 (all in orange). (TIF) [file pone.0026409.s002.tif]
